# Editorial: Mechanism of colonization and persistence of gut commensal microbiota and pathogens

**DOI:** 10.3389/fcimb.2023.1176453

**Published:** 2023-03-09

**Authors:** Pablo Castro-Córdova, Marjorie Pizarro-Guajardo, Alba Romero-Rodríguez

**Affiliations:** ^1^ Center of Interventional Medicine for Precision and Advanced Cellular Therapy (IMPACT), Santiago, Chile; ^2^ Laboratory of Nano-Regenerative Medicine, Centro de Investigación e Innovación Biomédica (CIIB), Faculty of Medicine, Universidad de los Andes, Santiago, Chile; ^3^ Department of Biology, Texas A&M University, College Station, TX, United States; ^4^ Departamento de Biología Molecular y Biotecnología, Instituto de Investigaciones Biomédicas, Universidad Nacional Autónoma de México, Ciudad de México, Mexico

**Keywords:** microbiota, gut colonization, bacterial persistence, gut pathogen, commensal microbiota

In the gastrointestinal tract, a diverse microbial community, including eukaryotes, bacteria, archaea, and bacteriophage, plays a role in human health and disease (Fan and Pedersen, 2021). To establish and colonize the human gut, several physiological and biological stressors along the digestive tract, such as bacteriophages (Federici et al., 2021), antimicrobial peptides (Mookherjee et al., 2020), bile acids (Collins et al., 2022), and antibiotic intake (Maier et al., 2021), must be overcome by commensal and gut bacteria. In addition, commensal and pathogenic microorganisms have mechanisms to survive and persist in an unfavorable environment, including sporulation, persister cells, viable non-cultivable cells, the inclusion of specific metabolic routes, specific factors, and genetic changes (Dworkin and Losick, 2001). Furthermore, the microbiota is challenged by the intrinsic and extrinsic factors of their hosts, such as diet, age, communicable and non-communicable diseases, and even vaccination. Consequently, adaptation and survival mechanisms of the microbiota play a critical role in gut microbiota modeling. These mechanisms employed to endure and survive in the colonic tract have been explored mainly in pathogens, especially those contributing to the relapse or recurrence of disease (Colangeli et al., 2020; Castro-Córdova et al., 2021). Their role in gut commensals is about to be dissected.

In this Research Topic, we aimed to cover recent and novel research about mechanisms for colonization and persistence employed by commensal and pathogenic bacteria to help us understand which are the factors that shape gut microbiota organization. In addition, we expect to know why dysbiosis is increased in aging and which could be the factors that promote these changes.

The current Research Topic contains three original articles and one mini-review aiming to understand the host-microbial factors or interactions involved in the establishment and maintenance of a healthy microbiota.

The lifespan of different gut microbiota species is determined by age-related changes. Yan et al. studied gut microbiome changes across age groups, eating habits, and metabolism and observed an increase in a group of bacteria in older Chinese people (*Bacteroides fragilis*, *Bifidobacterium longum*, *Clostridium bolteae*, *Escherichia coli*, *Klebsiella pneumoniae*, and *Parabacteroides merdae*) compared with young individuals, that correlated with an enrichment in metabolic pathway activation related to lipopolysaccharide (LPS) biosynthesis and degradation of short-chain fatty acids (SCFAs). On the other hand, Vidal-Veuthey et al., describe the role of proteins secreted by the gut microbiota and its positive or negative influence on the host. Altogether, these suggest that the metabolic pathways associated with the gut microbiota can be related to the immune status and inflammation of older adults, and the discovery of new microbiota-secreted proteins with a positive impact on the host could have therapeutic potential to be used in certain diseases.

In general, the presence and abundance of a bacteria specie have been linked to the protection or risk of a determinate disease. Here, Vega et al. examine whether the changes in the abundance of a determinate microbiome community have been coined as biomarkers for intestinal inflammatory diseases (IBD). By studying the public data on microbiome of patients with IBD, the authors observed that a group of bacteria (Fusobacterium, Streptococcus, and Escherichia/Shigella) were clearly abundant in IBD microbiome. They discuss the importance of redefine microbiome biomarkers by including the intra-taxa diversity, environmental and genetic factors present in the host to explore new candidates for the next generation of probiotics.

Adeno-associated virus vectors are a promising biomedical technology due that can transduce peripheral, autonomic, and enteric nervous systems. Ma et al., describe the effects of transduction with intraperitoneally administered adeno-associated virus 9 (AAV9) vector in the fecal microbiota in rats, where they observed an efficient transduction in the colon and changes in the microbial diversity, as well as upregulation of anaerobic gut microbiota.

To conclude, the gut microbiota changes constantly and is affected by several factors, including diet, diseases, genetic factors, microbial interaction, and infection/diseases such as inflammatory diseases or AAV infection. However, the influence of the microbiota changes, affecting the metabolism and the secretion of bacterial proteins and its effects in the host, is still to be understood. Indeed, targeting the microbiome and its metabolism is an attractive target for the development of new probiotics for better aging and to combat diseases.

## Author contributions

PC-C, MP-G, and AR-R contributed to the conception and design of the manuscript. PC-C wrote the first draft of the manuscript. All authors contributed to the article and approved the submitted version.
